# Differential DNA Damage Response of Peripheral Blood Lymphocyte Populations

**DOI:** 10.3389/fimmu.2021.739675

**Published:** 2021-09-14

**Authors:** Kerstin Felgentreff, Catharina Schuetz, Ulrich Baumann, Christian Klemann, Dorothee Viemann, Simona Ursu, Eva-Maria Jacobsen, Klaus-Michael Debatin, Ansgar Schulz, Manfred Hoenig, Klaus Schwarz

**Affiliations:** ^1^Department of Pediatrics and Adolescent Medicine, University Medical Center Ulm, Ulm, Germany; ^2^Department of Pediatrics, Medical Faculty Carl Gustav Carus, Technische Universität Dresden, Dresden, Germany; ^3^Department of Pediatric Pulmonology, Allergy and Neonatology, Hannover Medical School, Hannover, Germany; ^4^Core Facility Cytometry, Ulm University Medical Faculty, Ulm, Germany; ^5^Institute for Transfusion Medicine, University Ulm, Ulm, Germany; ^6^The Institute for Clinical Transfusion Medicine and Immunogenetics Ulm, German Red Cross Blood Service Baden-Wuerttemberg - Hessen, Ulm, Germany

**Keywords:** DNA damage response, peripheral blood lymphocyte subsets, mass cytometry, ataxia telangiectasia, cell cycle

## Abstract

DNA damage occurs constantly in every cell triggered by endogenous processes of replication and metabolism, and external influences such as ionizing radiation and intercalating chemicals. Large sets of proteins are involved in sensing, stabilizing and repairing this damage including control of cell cycle and proliferation. Some of these factors are phosphorylated upon activation and can be used as biomarkers of DNA damage response (DDR) by flow and mass cytometry. Differential survival rates of lymphocyte subsets in response to DNA damage are well established, characterizing NK cells as most resistant and B cells as most sensitive to DNA damage. We investigated DDR to low dose gamma radiation (2Gy) in peripheral blood lymphocytes of 26 healthy donors and 3 patients with ataxia telangiectasia (AT) using mass cytometry. γH2AX, p-CHK2, p-ATM and p53 were analyzed as specific DDR biomarkers for functional readouts of DNA repair efficiency in combination with cell cycle and T, B and NK cell populations characterized by 20 surface markers. We identified significant differences in DDR among lymphocyte populations in healthy individuals. Whereas CD56^+^CD16^+^ NK cells showed a strong γH2AX response to low dose ionizing radiation, a reduced response rate could be observed in CD19^+^CD20^+^ B cells that was associated with reduced survival. Interestingly, γH2AX induction level correlated inversely with ATM-dependent p-CHK2 and p53 responses. Differential DDR could be further noticed in naïve compared to memory T and B cell subsets, characterized by reduced γH2AX, but increased p53 induction in naïve T cells. In contrast, DDR was abrogated in all lymphocyte populations of AT patients. Our results demonstrate differential DDR capacities in lymphocyte subsets that depend on maturation and correlate inversely with DNA damage-related survival. Importantly, DDR analysis of peripheral blood cells for diagnostic purposes should be stratified to lymphocyte subsets.

## Introduction

Cellular DNA damage is constantly ongoing in every cell. It can be caused by external factors such as ionizing (IR) or ultraviolet (UV) radiation, chemicals - including alkylating drugs used as anti-cancer therapy - or by endogenous factors such as replicative and metabolic stress leading to accumulation of reactive oxygen species (ROS) (1, 2). While these insults may result in both DNA single strand breaks (SSBs) and double strand breaks (DSBs), the latter are more critical in terms of cell survival and mutation probability. Importantly, DNA DSBs are also physiologically introduced in the T cell receptor (TCR) and immunoglobulin (Ig) genes during V(D)J recombination and class switch recombination of developing lymphocytes (3). The cellular integrity relies on a complex repair system that ensures immediate sensing and efficient repair to protect the DNA from any persisting damage, known as DNA damage response (DDR). In case of failure of these mechanisms, apoptosis, senescence, or introduction of chromosomal breaks and mutations potentially leading to neoplastic transformation are the consequences (4).

DNA DSBs can be repaired by at least two major pathways in mammalian cells: non-homologous end-joining (NHEJ), which operates throughout the cell cycle, but is primarily required in the G0/G1 phase, and homologous recombination (HR), which relies on the presence of a sister chromatid in late S/G2 phase (1). Additionally, DSB are repaired by alternative end-joining pathways (A-EJ) including microhomology-mediated end-joining (MHMEJ) and single strand annealing (SSA) (5). In NHEJ mediated repair, Ku70 and Ku80 are the essential sensors of free DNA ends. They bind and stabilize the DNA, and further recruit the catalytic subunit of the DNA-dependent protein kinase (DNA-PKcs). Together they form an active serine/threonine DNA-PK holoenzyme that belongs to the phosphatidylinositol 3-kinase-related kinases (PIKKs) family. This complex recruits the endonuclease ARTEMIS, which processes modified DNA ends with overhangs, the XRCC4/Ligase 4 heterodimer, XLF and PAXX protein to complete the repair process (6). Genetic defects in NHEJ result in severe combined immunodeficiency with lack of T and B lymphocytes, but also increased cellular radiation sensitivity (RS-SCID) (7).

A major sensor of DNA DSB is the MRN complex formed by MRE11, RAD50 and NBS1 that hooks free DNA ends and activates the protein kinase ATM (ataxia telangiectasia mutated) (8). In addition, the kinase ATR (ataxia telangiectasia and Rad3 related) responds to single strand breaks (SSBs) including lesions caused by replication stress (9). ATM and ATR are both central player in regulation of cell cycle checkpoints, cell survival and DNA repair (10). Together with DNA-PKcs, they belong to the PIKK family and activate damage-specific signaling cascades of multiple different effector proteins through direct phosphorylation, such as cell cycle proteins CHK1 and CHK2. Whereas CHK1 is a target of ATR, CHK2 is predominantly activated by ATM, which promotes an arrest of cell cycle progression at the G1-S, and G2-M cell-cycle checkpoints in order to enable DNA repair before replication or mitoses ensues (2). Unrepaired DNA damage induces permanent cell cycle arrest (senescence) or apoptosis, which is balanced by the tumor suppressor protein p53 (11). In parallel, ATM/ATR signaling enhances DNA repair by recruitment of DNA repair factors through modulated phosphorylation. In particular, the histone protein H2AX is phosphorylated at serine 139 (γH2AX) by PIKKs such as ATR, ATM and DNA-PKcs in response to DNA DSBs (12). Following efficient repair, γH2AX is de-phosphorylated in a kinetic related to DNA repair efficiency (13, 14).

Genetic defects in *ATM* lead to ataxia telangiectasia (AT) presenting with combined immunodeficiency, genomic instability with predisposition to cancer, severe cellular sensitivity to IR, and cerebellar degeneration (15). Individuals affected by inborn DNA repair defects, including AT, often present with lymphopenia, predominantly reduced naïve T cells and B cells, and hypogammaglobinemia (16). In order to provide adequate treatment to these patients and to minimize DNA damaging diagnostic and treatment procedures, early diagnosis and classification of these diseases is highly important to affected individuals. DDR factors phosphorylated in a kinetic fashion in response to DNA damage can be used as biomarkers to assess DNA repair capacities (17–20).

Differential survival of lymphocyte subsets in response to DNA damage has been studied by many groups (21–25). Whereas NK cells are more resistant to DNA damage than T lymphocytes, B cells seem to be the most sensitive lymphocytic population. Reduced DDR has been reported in resting T cells that activate the ATM pathway in response to DNA DSB but fail to form γH2AX foci and undergo apoptosis more extensively than proliferating T cells (26).

In this study, we investigated the ATM-dependent DDR by mass cytometry using γH2AX, p-ATM, p-CHK2 and p53 as biomarkers in peripheral blood lymphocyte subsets. T, B and NK cell populations were characterized by 20 surface markers, and DDR capacities were stratified to these lymphocyte populations. We observed increased γH2AX formation in NK cells and diminished H2AX phosphorylation in B lymphocytes that correlated inversely with p53 induction and reported survival responses. Similar results were obtained for CD4^+^ and CD8^+^ naïve versus memory T cells. Furthermore, we elucidated that proliferation and cell cycle impacted on DDR intensity, but not on differential DDR capacity in lymphocyte subsets.

## Materials and Methods

### Samples, Cell Culture, and DNA Damage Induction

Study of patients was approved by the ethical review boards of Ulm University (407/16), Technical University of Dresden (TUD) (BO-EK-213052020), and Hannover Medical School (9492-BO-K-2020), and patients and parents gave informed consent to this investigation. Peripheral blood mononuclear lymphocytes (PBMC) samples of anonymized buffy coat donors were used as controls. PBMCs were isolated from 26 healthy donors and 3 patients diagnosed with AT using Ficoll Paque Plus (GE healthcare) according to the manufacturer’s instructions. Cells were frozen in 50% fetal calf serum (FCS) (Biowhittacker and PAN Biotech), 40% RPMI 1640 media (Gibco) and 10% DMSO (Roth), and stored in liquid nitrogen.

PBMCs were thawed and plated in RPMI 1640 media (Gibco) supplemented with 15% FCS (Biowhittacker and PAN Biotech), 1% glutamine (Thermo Fisher Scientific), 1% non-essential amino acids (NEAA) (Thermo Fisher Scientific), 1% penicillin/streptavidin (Thermo Fisher Scientific), 100U/ml IL-2 (R&D Systems) at 1x10^6/ml in a 24-well culture dish (CellStar^®^, Greiner Bio-One) and incubated at 37°C 5% CO_2_ for 48h-96h.

After resting for 2-4d, 0.5-1x10^6 cells were used for each time point and treated with 2Gy of ionizing radiation or 100mJ/m^2^ UVC, respectively. An untreated sample served as negative control. Up to 4 donors were analyzed within one experiment.

### Viability and Surface Staining

Cells were stained with the viability marker Cell ID™ cisplatin (Fuidigm) and surface markers prior to fixation. Cells were pelleted (300g, 5min) in 5ml round bottom tubes (Folcon^®^, Corning), and resuspended in 800ml of 5µM Cell ID cisplatin solution diluted in PBS (Thermo Fisher Scientific). After incubation at RT for 5min, the reaction was stopped by adding 4ml staining buffer (PBS, 1% FCS, 2mM EDTA (Thermo Fisher Scientific). After centrifugation, the supernatant was removed completely, and cells were resuspended in 100µl staining buffer containing antibody mixes. The following surface antibodies were used at concentrations of 1µl/100µl staining buffer: Mouse anti-human CD45 (HI30)-154Sm (Fluidigm), anti-human CD3 (UCHT1)-170Er (Fluidigm), anti-human CD4 (SK3)-174Yb (Fluidigm), anti-Human CD8 (Sk1)-168Er (Fluidigm), anti-human CD45RA (HI100) 169Tm (Fluidigm), anti-human CD45RO (UCHL1) 164-Dy (Fluidigm), anti-human CD197/CCR7 (G053H7) 159Tb (Fluidigm), anti-human CD69 (FN50) 144Nd (Fluidigm), anti-human CD56 (HCD56) 176Yb (Fluidigm), anti-Human CD16 (3G8)-165Ho/Fluidigm, anti-human CD57 (HCD57) 163Dy (Fluidigm), anti-human CD19 (HIB19) 142Nd (Fluidigm), anti-human CD20 (2H7) 171Yb (Fluidigm), anti-human IgD (IA6-2) 146Nd (Fluidigm), anti-human IgM (MHM-88) 172Yb (Fluidigm), anti-human IgG kappa (MHK-49) 160Gd (Fluidigm), anti-human IgG lambda (MHL-38) 151Eu (Fluidigm), anti-human CD27 (L128) 158Gd (Fluidigm), anti-human CD38 (HIT2) 167Er (Fluidigm), anti-human CD21 (BL-13) 152Sm (Fluidigm). PBMCs were incubated in antibody mixes for 30min at RT, and subsequently washed once with 2ml/tube staining buffer.

### Fixation and Permeabilization

Cells were fixed in 5ml round bottom tubes 1h, 4h, 8h and 24h after DNA damage induction using the solution A of Fix&Perm (Thermo Fisher Scientific) diluted 1:1 with PBS. After incubation at RT for 10min, 2ml chilled methanol was added to each sample. Samples were stored at -20°C for at least 10min up to 1 week.

### Barcoding and Intranuclear Staining

All samples were barcoded using the Cell-ID™ 20-Plex PdBarcoding Kit (Fluidigm). After removing methanol and two washes with staining buffer, cells were incubated with 10µl Pd barcode 1-20 in 100µl staining buffer for 30min at RT. Samples were washed twice with 2ml staining buffer and all 5 time points (unirradiated, 1h, 4h, 8h, 24) of each donor were pooled in one sample. Subsequently, cells were stained in 50µl staining buffer supplemented with intranuclear antibodies anti-p histone H2A.X (Ser139) (JBW301) 147Sm (Fluidigm) (2µl/100µl), anti-human p53 (DO-7) 150Nd (Fluidigm) (3µl/100µl), anti-ki-67 (B56) 162Dy (Fluidigm) (1µl/sample), anti-phospho-CHK2 (Thr68) antibody (12-9508-42) (Thermo Fisher Scientific) (5µl/100µl) and purified anti-ATM phosphor (Ser1981) (Biolegend) (2µl/100µl) at RT for 1h. The p-ATM antibody was labeled with 141Pr using the Maxpar^®^ multimetal labeling kit (Fluidigm) according to manufacturer’s instructions. The p-CHK2 PE antibody was detected using the maxpar-ready purified anti-phycoerythrin (PE) antibody (Biolegend) labeled with 161Dy. After incubation with the intranuclear antibodies mentioned above, cells were washed twice and stained in 50µl staining buffer supplemented with secondary anti-PE 161Dy antibody (3µl/100µl) and incubated at RT for 1h. Following two washes, cells were fixed in 3ml/sample of 1,6% formaldehyde (Pierce™ 16% formaldehyde methanol-free, Thermo Fisher Scientific) diluted in PBS and incubated at 4°C over night.

### Sample Acquisition

The following day, cells were labeled with Cell-ID™ intercalator Ir (Fluidigm) by incubation in 250nM iridium solution diluted in PBS (3ml/sample) at RT for 1h. Following washes with PBS and deionized water, cells were resuspended in deionized water, counted and strained through a 35µm nylon mash filter cap (Falcon^®^, Corning). Samples were acquired on a Helios (a CyTOF system) mass cytometer (Fluidigm). Acquired data of each sample were debarcoded and saved as fcs files. Data are available at https://dataverse.harvard.edu/.

### Cell Cycle Studies

For cell cycle studies, Cell ID™ 127 IdU (Fluidigm) was added to 1x^10^6^ cells/1ml suspension to a final concentration of 50µM 30min before the viability staining was started. IdU is incorporated in the DNA of replicating cells and can be read in the iodine channel on the Helios cytometer. For differentiation of cells in the G0 and G1 cell cycle phases, the proliferation marker ki67 was included in the intracellular staining mix.

### Gating and Data Analysis

Data files were analyzed using FlowJo™ v10 and Cytobank Premium software. Gating strategies are shown in **Supplementary Figure 1**. Live cells were defined as cisplatin^low^, and discrimination of single cells was performed by using 191Ir and 193Ir. We defined the following populations of CD45^+^ lymphocytes: CD3^+^, CD3^+^CD4^+^, naïve CD4^+^ (CD3^+^CD4^+^CD45RA^+^CCR7^+^), central memory CD4^+^ (CD3^+^CD4^+^CD45RO^+^CCR7^+^), effector memory CD4^+^ (CD3^+^CD4^+^CD45RO^+^CCR7^-^), CD3^+^CD8^+^, naïve CD8^+^ (CD3^+^CD8^+^CD45RA^+^CCR7^+^), central memory CD8^+^ (CD3^+^CD8^+^CD45RO^+^CCR7^+^), effector memory CD8^+^ (CD3^+^CD8^+^CD45RO^+^CCR7^-^), CD3^-^CD56^high^CD16^-^, CD3^-^CD56^high^CD16^+^, CD3^-^CD56^dim^CD16^+^, CD56^bright^CD16^+^ and CD56^dim^CD16^+^ were further analyzed as CD57^+^/CD57^-^, CD3^-^CD19^+^/CD20^+^, naïve B (CD3^-^CD19^+^CD20^+^CD27^-^IgD^+^), memory B (CD3^-^CD19^+^CD20^+^CD27^+^), unswitched memory B (CD19^+^CD20^+^CD27^+^IgM^+^), class switched memory B IgGκ (CD19^+^CD20^+^CD27^+^IgM^-^IgGκ^+^), class switched memory B IgGΛ (CD19^+^CD20^+^CD27^+^IgM^-^IgGΛ^+^), marginal zone (MZ)-like B (CD19^+^CD20^+^CD27^+^IgM^+^IgD^+^), transitional B (CD19^+^CD20^+^IgM^++^CD38^++^), IgM only B (CD19^+^CD20^+^CD27^+^IgM^+^IgD^-^), atypical memory B (CD19^+^CD20^+^CD27^-^IgM^-^IgD^-^) unswitched plasmablasts (CD19^+^CD20^-^CD27^+^CD38^+^IgM^+^), class switched plasmablasts (CD19^+^CD20^-^CD27^+^CD38^+^IgM^-^), and CD21^low^CD38^low^ B (CD19^+^CD20^+^CD21^low^CD38^low^) (27). Geometric mean fluorescence intensities (MFI) of γH2AX, p-ATM, p-CHK2, and p53, were calculated for each lymphocyte subset. Furthermore, cell cycle was analyzed by discrimination between ki67^+^IdU^+^ (S phase), ki67^+^IdU^-^ (G1 phase), and ki67^-^IdU^-^ (G0 phase) cells. Expression of an intranuclear marker was further analyzed in all cell cycle phases of all populations.

Premium Cytobank was used to generate t-SNE plots (t-distributed neighbor embedding) applying the same gates as used for statistical analysis. Intensities of nuclear markers (Z channel) are shown for all live/single/CD45^+^ cells. Distribution of lymphocyte populations are shown in key figures.

### Statistical Analysis

In order to compare MFIs of DDR markers generated from separate experiments, fold inductions of γH2AX, p-ATM, p-CHK2 and p53 were calculated by normalizing on MFIs of untreated samples. Of note, untreated samples and all time points of treated samples from one donor were pooled and stained in one tube.

Statistical analysis and generation of graphs was performed using graph pad prism v9 software. Statistical significance of DDR marker expression calculated by MFIs between different lymphocyte subsets was calculated by 2-way ANOVA and Turkey’s multiple comparison test. Side by side comparisons of samples that were treated with and without IL-2, as well as ki67^+^ and ki67^-^ samples, were analyzed using Šídák’s test. Cell counts of IL-2 treated samples of various populations were compared to non-treated samples using student’s T test. P-values ≤0.05 were considered significant (*p ≤ 0.05, **p ≤ 0.01, ***p ≤ 0.001, ****p ≤ 0.0001).

## Results

### DNA Damage Response to Ionizing Radiation Differs Among T, B, and NK Lymphocytes

Frozen PBMCs of 26 healthy donors were thawed and cultured in RPMI media and 100U/ml IL-2 for 4d in order to recover from DNA damage. Following low dose ionizing radiation (IR) of 2Gy, cells were fixed after 1h, 4h, 8h and 24h. DDR markers γH2AX, p-ATM, p-CHK2, and p53 were assessed in T, B, and NK lymphocyte subsets by mass cytometry. All time points obtained from one individual were pooled to be stained with all intranuclear markers in one tube. In order to compare results from different individuals and experiments, the fold induction of DDR markers was calculated based on untreated samples. While induction of γH2AX, p-ATM, and p-CHK2 could be observed early and decreased over time once DNA damage was repaired, p53 peaked at 8h after IR.

**Figure 1:**
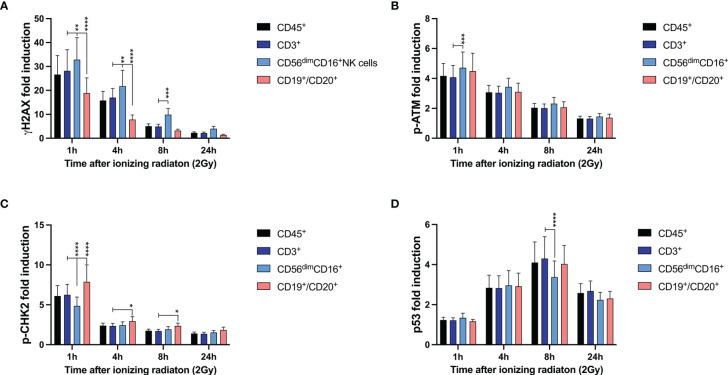
DNA damage response to ionizing radiation differs among T, B, and NK lymphocytes. Peripheral blood mononuclear cells (PBMCs) from 26 healthy donors were irradiated with 2Gy and fixed at indicated time points. Surface markers of lymphocyte subsets and intranuclear DDR biomarkers were analyzed by mass cytometry. Induction of γH2AX **(A)**, p-ATM **(B)**, p-CHK2 **(C)**, and p53 **(D)** were calculated in CD45^+^ lymphocytes, CD45^+^CD3^+^ T cells, CD45^+^CD56^dim^CD16^+^ NK cells and CD45^+^CD19^+^CD20^+^ B cells based on mean fluorescence intensities normalized on unirradiated samples. Bars represent mean values; error bars represent standard deviations. Significance is shown for NK and B lymphocytes in comparison to T cells (*p ≤ 0.05, **p ≤ 0.01, ***p ≤ 0.001, ****p ≤ 0.0001).

Compared to CD3^+^ T cells, CD3^-^CD56^dim^CD16^+^ NK cells showed significantly increased γH2AX induction 1h, 4h and 8h after IR, whereas significantly lower γH2AX level were detected in CD3^-^CD19^+^CD20^+^ B cells (**Figure 1**). This correlated with a slightly elevated p-ATM response in NK cells, that did not differ between T and B lymphocytes, and reduced p53 induction in CD3^-^CD56^dim^CD16^+^ NK cells 8h after IR. Interestingly, p-CHK2 response was observed predominantly in B cells, and to a much lesser extend in NK and T lymphocytes.

**Figure 2:**
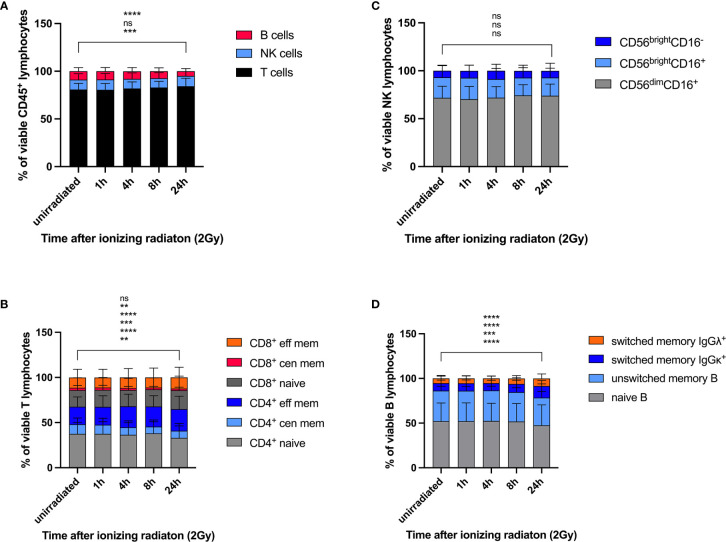
Lymphocyte subsets show differential survival response rates to ionizing radiation. PBMCs obtained from 26 healthy donors were irradiated with 2Gy and fixed at indicated time points. Cell counts of viable (cisplatin^-^) T, NK, and B lymphocytes **(A)**, naïve and memory CD4^+^ and CD8^+^ T cell subsets **(B)**, CD56^bright^CD16^-^, CD56^bright^CD16^+^, CD56^dim^CD16^+^ NK cell subsets **(C)**, and naïve and memory B cell populations **(D)** were compared at each time point following radiation. Statistical significance was calculated for each lymphocyte population using Turkey’s multiple comparison test and is shown for unirradiated lymphocytes *vs.* lymphocytes 24h after radiation (ns, not significant, **p ≤ 0.01, ***p ≤ 0.001, ****p ≤ 0.0001).

Viability of lymphocyte subsets in response to IR was studied according to Cell-ID™ cisplatin staining. Proportions of T, NK, and B lymphocytes were calculated as percentages of the viable (cisplatin^-^) CD45^+^ lymphocyte population before and 1h, 4h, 8h, 24h after IR. Whereas the proportion of unirradiated B cells significantly declined 24h after IR (mean 8.1% to 4.5%), percentages of T cells compensatory increased (mean 75.5% to 77.2%), and the proportion of NK cells did not change (mean 7.0% to 7.2%) (**Figure 2A**). These results indicate a poorer survival rate of B lymphocytes in response to ionizing radiation compared to T and NK cell populations.

Our results demonstrate differential DDR among lymphocyte subsets, whereas increased γH2AX levels correlate with reduced p-CHK2 and p53 response. In B lymphocytes, a reduced DDR as reflected by γH2AX induction was associated with reduced survival rates compared to T and NK lymphocytes.

### DDR in Naïve Lymphocyte Subsets Is Differential to Mature Memory Populations

**Figure 3:**
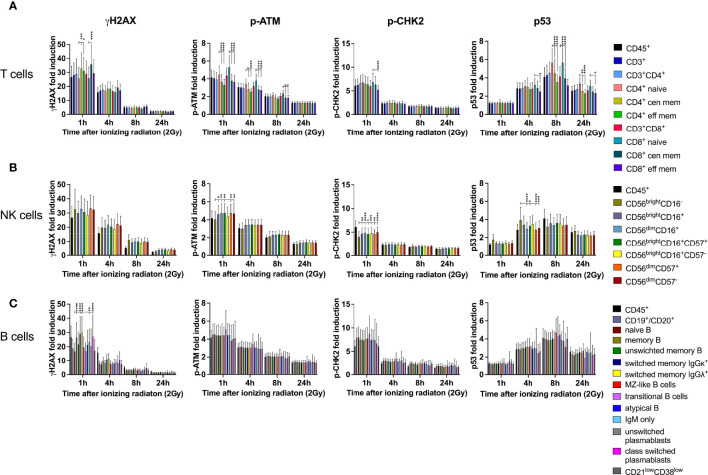
DDR in naïve lymphocyte subsets is differential to mature memory populations. PBMCs obtained from 26 healthy donors were irradiated with 2Gy and fixed at indicated time points. Surface markers of lymphocyte subsets and intranuclear DDR biomarkers were assessed by mass cytometry. Fold inductions of γH2AX, p-ATM, p-CHK2, and p53 were calculated in CD45^+^ lymphocyte subsets, based on mean fluorescence intensities normalized on unirradiated samples. T cell subsets were characterized as CD3^+^, CD3^+^CD4^+^, CD3^+^CD8^+^, CD45RA^+^CCR7^+^ (naïve CD4/CD8), CD45RO^+^CCR7^+/-^ (central and effector memory CD4/CD8) **(A)**. NK lymphocyte subsets were defined as CD3^-^CD56^bright^CD16^-^, CD3^-^CD56^bright^CD16^+^, CD3^-^CD56^dim^CD16^+^ **(B)**, which were further stratified to CD57 expression on CD56^bright^CD16^+^ and CD56^dim^CD16^+^ subsets. CD3^-^ B lymphocytes were characterized as CD19^+^CD20^+^, CD27^-^IgD^+^(naïve B), CD27^+^ (memory B), CD27^+^IgM^+^ (unswitched memory B) CD27^+^IgM^-^IgGκ^+^ (class switched memory B IgGκ), CD27^+^IgM^-^IgGΛ^+^ (class switched memory B IgGΛ), CD27^+^IgM^+^IgD^+^ (Marginal Zone (MZ)-like B), IgM^++^CD38^++^ (transitional B), CD27^+^IgM^+^IgD^-^ (IgM only B), CD27^-^IgM^-^IgD^-^ (atypical memory B), CD19^+^CD20^-^CD27^+^CD38^+^IgM^+^ (unswitched plasmablasts), CD19^+^CD20^-^CD27^+^CD38^+^IgM^-^ (class switched plasmablasts), and CD21^low^CD38^low^ B cells. **(C)** Bars represent mean values of fold induction; error bars represent standard deviations. Significance is shown for naïve T and B cell subsets compared to memory subsets, and immature CD56^bright^CD16^-^ to mature CD56^bright^CD16^+^ and CD56^dim^CD16^+^ NK lymphocytes (*p ≤ 0.05, **p ≤ 0.01, ***p ≤ 0.001, ****p ≤ 0.0001).

DDR was investigated in all T, B and NK lymphocyte subsets included in this panel. Naïve CD3^+^CD4^+^ and CD3^+^CD8^+^ T cells responded to low dose IR with lower γH2AX induction, but enhanced p-ATM and p53 response compared to central and effector memory cells (**Figure 3A**). Of note, similar results were obtained for CD4^+^ as well as CD8^+^ T cells, and DDR was stronger in central than in effector memory T cells. Induction of p-CHK2 was only slightly reduced in memory subsets, although significantly for CD8^+^ central memory. Expression of the activation marker CD69 did not impact on DDR (data not shown). In addition to DDR, we analyzed viability of naïve and memory CD4^+^ and CD8^+^ T cell subsets by calculating their proportions of cisplatin^-^ CD45^+^CD3^+^ T cells and their changes over time in response to IR. We observed significant reduction of naïve (mean 37.6% to 33.1%) and central memory (mean 10.5 to 7.8%) CD4^+^ T cells 24h after IR and compensatory increase of effector memory CD4^+^ cells (**Figure 2B**). On the contrary, proportions of viable naïve CD8^+^ T lymphocytes increased (mean 36.0% to 39.5%) to the costs of central memory CD8^+^.

Despite γH2AX induction did not differ between immature CD56^bright^CD16^-^, CD56^bright^CD16^+^ and mature CD56^dim^CD16^+^ NK cell subsets, p-ATM and p-CHK2 expression was significantly increased in CD56^bright^CD16^+^ and CD56^dim^CD16^+^ populations compared to CD56^bright^CD16^-^ (**Figure 3B**). CD56^bright^CD16^-^ NK cells are mostly located in secondary lymphoid organs, where they mature into CD56^dim^CD16^+^ NK cells, the most abundant population in peripheral blood. Overall, expression of all DDR markers varied among individuals due to variable distribution of the three NK cell subsets analyzed. Expression of the senescence marker CD57, which we studied on CD56^bright^CD16^+^ and CD56^dim^CD16^+^ NK cells, did not impact on DDR. CD56^bright^CD16^-^ cells did not express CD57. Survival of CD56^bright^CD16^-^, CD56^bright^CD16^+^ and CD56^dim^CD16^+^ NK cell subsets was not affected by IR, as their proportions did not change (**Figure 2C**).

Whereas naïve B lymphocytes were characterized by reduced γH2AX compared to memory B cells, including unswitched and class switched memory B cells, IgM only B cells and class-switched plasmablasts, there were no differences among the other DDR markers investigated on B cell subsets (**Figure 3C**). Analysis of proportional changes of cell counts revealed significant decrease of naïve (mean 52.3% to 47.5%) and unswitched memory B cells (mean 34.1% to 31.1%) 24h after IR (**Figure 2D**).

### Stimulation With IL-2 Does Not Impact on Differential DDR Capacities in Lymphocyte Subsets

Since PBMCs were cultured with IL-2 to enable recovery from cellular stress for several days before they were irradiated, we investigated whether IL-2 had an impact on DDR. PBMCs of six healthy donors were cultured either without or in presence of 100U/ml IL-2 in RPMI media for 4d before low dose IR (2Gy). Comparison of lymphocyte subset proportions revealed significantly increasing numbers of NK cells in response to IL-2, whereas T and B lymphocyte counts were not affected (**Supplementary Figures 2A, B, D**). Within the NK cell population, an increase of CD56^bright^CD16^-/+^ populations could be observed, and the proportion of CD56^dim^CD16^+^ cells was significantly enlarged in unstimulated samples (**Supplementary Figure 2C**).

**Figure 4:**
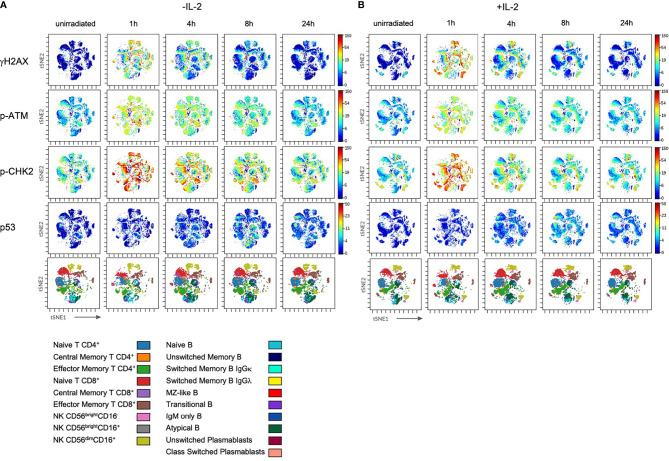
Stimulation with IL-2 does not impact on DDR in lymphocyte subsets. PBMCs of healthy donors were cultured in RPMI media without (w/o) supplementation of IL-2 **(A)** and in presence of 100U/ml human IL-2 **(B)** for 48h. Subsequently, cells were irradiated with 2Gy and fixed at indicated time points. Shown are tSNE Plots demonstrating expression level of DDR markers γH2AX, p-ATM, p-CHK2 and p53 on all populations analyzed at indicated time points. Scale bars on the right-hand side of each panel indicate intensities of DDR markers. The bottom panel of **(A**, **B)**, respectively, represent lymphocyte populations that are color coded by the legend underneath. This figure demonstrates one representative experiment of one donor out of six.

**Figure 5:**
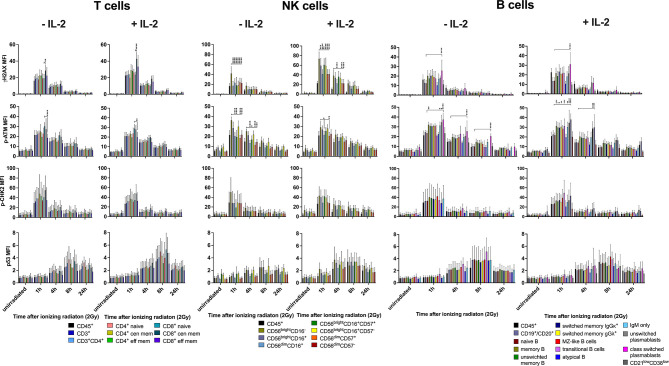
Differential DDR in lymphocyte subsets is independent from IL-2 stimulation. PBMCs of 6 healthy donors were cultured in RPMI media without and in presence of 100U/ml human IL-2 for 48h. Cells were irradiated with 2Gy and fixed at indicated time points. Mean fluorescence intensities (MFI) of DDR markers γH2AX, p-ATM, p-CHK2 and p53 are shown in T, NK and B lymphocyte subsets of unirradiated lymphocytes and 1h, 4h, 8h, 24h following IR with 2Gy (*p ≤ 0.05, **p ≤ 0.01, ***p ≤ 0.001, ****p ≤ 0.0001).

IL-2 stimulation led to stronger IR-related γH2AX and p53 induction in T and NK cell subsets, whereas p-CHK2 was reduced compared to untreated cells (**Figures 4** and **5**). No differences were observed regarding the response of p-ATM. Of note, differential DDR capacities between naïve and memory subsets could be observed regardless of the γH2AX, p-CHK2 and p53 induction level. As expected, IL-2 had no effect on γH2AX induction in B cell subsets. However, p-CHK2 and p53 were increasingly upregulated in untreated B lymphocytes, which correlated with their cell cycle profile. A side-by-side comparison of DDR in IL-2 treated and non-treated samples showed significant differences of γH2AX induction in NK cell subsets, however, not in other populations (**Supplementary Figures 3**–**5**).

In congruence with results obtained from IL-2 treated samples, IR affected survival of B lymphocytes, whereas proportions of other lymphocyte subsets did not change significantly, except for naïve CD8^+^ T and CD56^dim^CD16^+^ NK cells (**Supplementary Figures 6A–D**).

**Figure 6:**
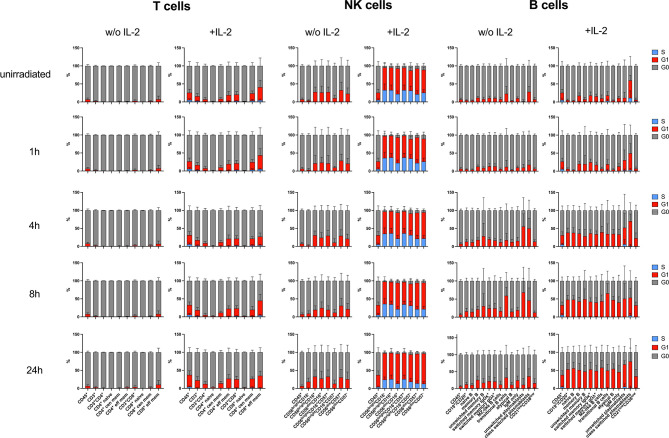
Stimulation with IL-2 impacts on cell cycle of lymphocyte subsets. PBMCs of six healthy donors were cultured in RPMI media without (w/o) supplementation of IL-2 and in presence of 100U/ml human IL-2 for 48h. Cells were irradiated with 2Gy, and 30min before fixation at indicated time points, IdU was added to cell culture to be incorporated in the DNA of replicating cells. Cell cycle was assessed by mass cytometry using ki67 and IdU to distinguish G0 (ki67^-^IdU^-^), G1 (ki67^+^IdU^-^) and S (ki67^+^IdU^+^) cell cycle phases. Bars represent mean percentages of cell cycle phases G0 (grey), G1 (red), and S (blue) in T, NK and B lymphocyte subsets of unirradiated lymphocytes and 1h, 4h, 8h, 24h after IR with 2Gy. CD45^+^ lymphocytes are shown in each panel as internal control. Error bars represent standard deviations.

In order to study the impact of IL-2 on cell cycle in lymphocyte subsets investigated, the proliferation marker ki67 and IdU were used. IdU was added to cell cultures 30min before fixation and incorporated in the DNA of replicating cells in the S phase of the cell cycle. Ki67 discriminates cells in G0 and G1 cell cycle phases. IL-2 had only minor effects on T cell subsets activating up to 40% of ki67^+^ cells in G1 phase (**Figure 6**). In contrast, NK cell subsets replicated in response to IL-2 stimulation. Whereas up to 60% of CD56^bright^CD16^-^ NK cells could be detected in S phase of the cell cycle, only up to 33% of CD56^dim^CD16^+^ NK cells were proliferating. The replication rate decreased 24h after IR. B lymphocyte populations did not proliferate in response to IL-2, but transitioned to G1 (ki67^+^) 4h-24h after IR. This effect could be observed in presence and absence of IL-2, although it was enhanced by IL-2. It is known that recombinant IL-2 can promote B cell proliferation mediated through Tac expressed on both activated T and B lymphocytes (28). What is driving B cell activation in our assay needs to be addressed in future studies.

In summary, these results show that IL-2 impacts on proliferation of T and NK cells, although only the latter are replicating, which increases DNA DSB breaks and therefor γH2AX level. However, DDR capacities of T, B, and NK lymphocyte subsets are not altered by stimulation with IL-2.

### Cell Proliferation Impacts on DDR, but Is Irrespective of Differential DDR Capacities of Lymphocyte Subsets

It is well established that cell proliferation impacts on DDR and sensitivity to DNA damage. We therefor analyzed induction of γH2AX, p-CHK2, p-ATM and p53 in response to IR of 2Gy in ki67^+^ and ki67^-^ subsets of 8 healthy donor PBMCs cultured in RPMI supplemented with 100U/ml IL-2 (**Supplementary Figure 7**). Regardless of proliferation indicated by ki67 expression, memory CD4^+^ and CD8^+^ T cells showed higher induction levels of γH2AX than naïve T cells, and a significantly reduced p53 response. In particular, ki67^+^ NK cell subsets differed in γH2AX and p53 induction, which was significantly increased in CD56^bright^CD16^-^ compared to mature NK cells. Ki67^+^ and ki67^-^ B cell subsets responded in similar ways to IR. A side-by-side comparison of DDR induction in ki67^+^ and ki67^-^ cells revealed significant differences regarding γH2AX, p-ATM and p53 in CD56^bright^CD16^-^, CD56^bright^CD16^+^ and CD56^dim^CD16^+^ NK cell subsets, however not in T and B lymphocytes (**Supplementary Figure 8**).

In summary, proliferation characterized by ki67 expression, did not alter differential DDR of lymphocyte subsets.

### DDR Capacities to UVC Exposure Are Distinct to IR

**Figure 7:**
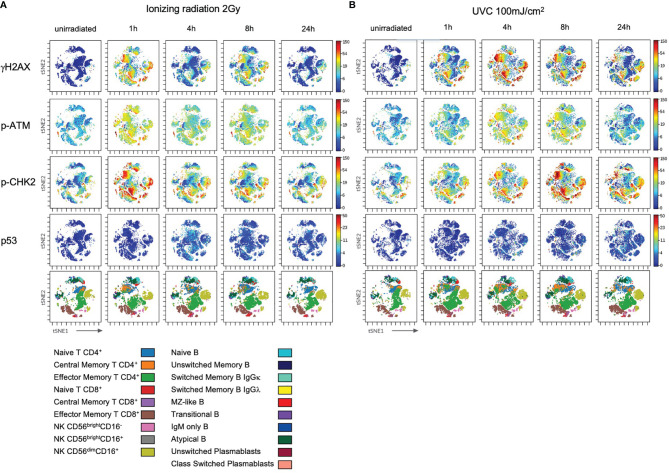
DDR to ultraviolet C radiation is differential in lymphocyte subsets. PBMCs of healthy donors were thawed and cultured in RPMI media supplemented with 100U/ml human IL-2 for 96h before ionizing radiation with 2Gy **(A)** and UVC radiation with 100mJ/m^2^ **(B)**. Cells were fixed unirradiated, and 1h, 4h, 8h, 24h after radiation. Shown are tSNE Plots demonstrating expression level of DDR markers γH2AX, p-ATM, p-CHK2 and p53 on all populations at indicated time points. Scale bars on the right-hand side of each panel indicate intensities of DDR markers. The bottom panel of **(A**, **B)**, respectively, represent populations color coded by the legend underneath. Shown is one representative experiment obtained from one donor out of eight.

**Figure 8:**
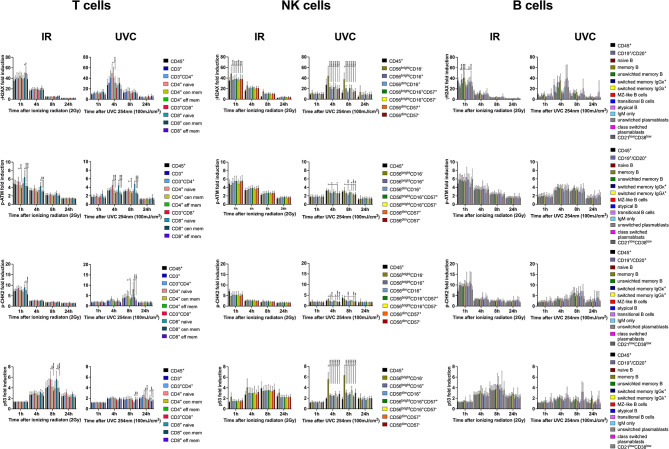
DDR of PBMCs to IR is differential to DDR induced by UVC. PBMCs obtained from 8 healthy donors were irradiated with 2Gy or 100mJ/m^2^ UVC, respectively. Surface markers of lymphocyte subsets and intranuclear DDR biomarkers were analyzed by mass cytometry. Fold inductions of γH2AX, p-ATM, p-CHK2, and p53 were calculated in CD45^+^ lymphocyte subsets, based on mean fluorescence intensities normalized on unirradiated samples. Bars indicate mean values of fold induction; error bars represent standard deviations. Significance is shown for naïve T and B cell subsets compared to memory subsets, and immature CD56^bright^CD16^-^ to mature CD56^dim^CD16^+^ NK lymphocytes (*p ≤ 0.05, **p ≤ 0.01, ***p ≤ 0.001, ****p ≤ 0.0001).

In addition to low dose IR, we investigated DDR in response to UVC exposure (100mJ/cm^2^) in PBMCs obtained from 8 healthy donors. In contrast to response to IR, γH2AX, p-ATM, and p-CHK2 peaked around 4h-8h after UVC radiation (**Figures 7** and **8**). We observed increased γH2AX response in CD4^+^ compared to CD8^+^ T cells, including naïve CD4^+^. P-ATM and p-CHK2 response was significantly increased in naïve versus memory subsets (**Figure 8**). P53 was less induced in response to UVC without significant differences among T lymphocyte populations. Interestingly, DDR to DNA damage induced by IR compared to UVC was most differential in NK cell subsets. CD56^bright^CD16^-^ NK cells showed strong γH2AX, p53, and to a lesser extend p-CHK2 and p-ATM inductions compared to CD56^bright^CD16^+^ and CD56^dim^CD16^+^ NK cell populations in response to UVC. Naïve B lymphocytes presented with slightly reduced γH2AX levels, as observed in response to IR. Other response markers were not altered in B cell subsets.

Like what observed in lymphocytes exposed to IR, UVC exposure impacted predominantly on survival of B cells (**Supplementary Figure 9A**), and survival of naïve B cells in particular (**Supplementary Figure 9D**). Furthermore, proportions of naïve CD8^+^ T and CD56^bright^CD16^+^ NK cells were altered 24h after IR (**Supplementary Figures 9B, C**).

Whereas DDR was similar among ki67^+^ and ki67^-^ T and B lymphocytes exposed to 100mJ/m^2^ UVC, diminished DDR of γH2AX and p53, could be observed in ki67^-^ NK cell subsets (**Supplementary Figure 10**). γH2AX and p53 were significantly increased in ki67^+^ CD56^bright^CD16^-^ compared to CD56^dim^CD16^+^ subsets at 4h and 8h after exposure. In contrast, p-ATM response was diminished in ki67^-^ CD56^dim^CD16^+^ NK lymphocytes. However, 80-99% of NK cell subsets expressed ki67 in response to IL-2 treatment, which complicates analysis of underrepresented ki67^-^ cells. A side-by-side comparison of DDR induction in ki67^+^ and ki67^-^ cells showed significantly increased induction of γH2AX and p53 in NK cells, but not in T and B lymphocyte subsets (**Supplementary Figure 11**).

Cell cycle studies revealed few differences among IR and UVC treated PBMCs (**Supplementary Figure 12**). The activating effect of DNA damage on B cell subsets that transitioned from ki67^-^ (G0) to ki67^+^ (G1) after 4h could only be observed in response to IR, but not UVC. Interestingly, a population of max. 22% (CD4 naïve) ki67^-^IdU^+^ cells could be observed 4h after UVC exposure in T, B and NK lymphocytes. Du Manoir et al. (29) described ki67^low^ BrdU^+^ cells in a transient, quiescent stage that did not progress to mitosis. These fractions are most sensitive to growth factors. According to these previous reports, these populations most likely stopped proliferation in response to UVC exposure but did not yet induce apoptosis. However, different ki67 expression among lymphocyte subsets depended on IL-2, but not DNA damage.

These results confirm that different DDR capacities are independent from the type of DNA damage but specific to lymphocyte subsets.

### DDR Is Abrogated in Patients With Ataxia Telangiectasia

ATM is the major PIKK kinase that phosphorylates H2AX, CHK2 and p53. However, also ATR and DNA-PKcs can catalyze H2AX activation. To investigate dependence of γH2AX, p-ATM, p-CHK2 and p53 activation to ATM function, we included three AT patients in our study. DDR in CD45^+^ lymphocytes, CD3^+^ T cell, CD3^-^CD56^dim^CD16^+^ NK cell and CD3^-^CD19^+^CD20^+^ B cell subsets was severely diminished in patients compared to 26 healthy controls. Although B cell counts severely diminished 24h after IR (mean 8.7% to 2.8%), this was not significant given the small sample size (**Supplementary Figure 13A**). Proportions of T lymphocytes were altered in AT patients compared to controls with reduced naïve CD4^+^ and CD8^+^ T cells that further declined in response to IR, although not significantly (**Supplementary Figures 13B–D**).

We further studied DDR distributed to lymphocyte subsets of AT patients. Apart from partial activation of γH2AX in effector memory T cells and CD56^bright^CD16^-^ and CD56^bright^CD16^+^ NK lymphocytes, no differential DDR could be observed among lymphocyte subsets (**Supplementary Figures 14**, **15**). Phosphorylation of H2AX can be best compensated by other PIKK enzymes, such as DNA-PKcs and ATR. Nevertheless, ATM is the major factor activating H2AX.

**Figure 9:**
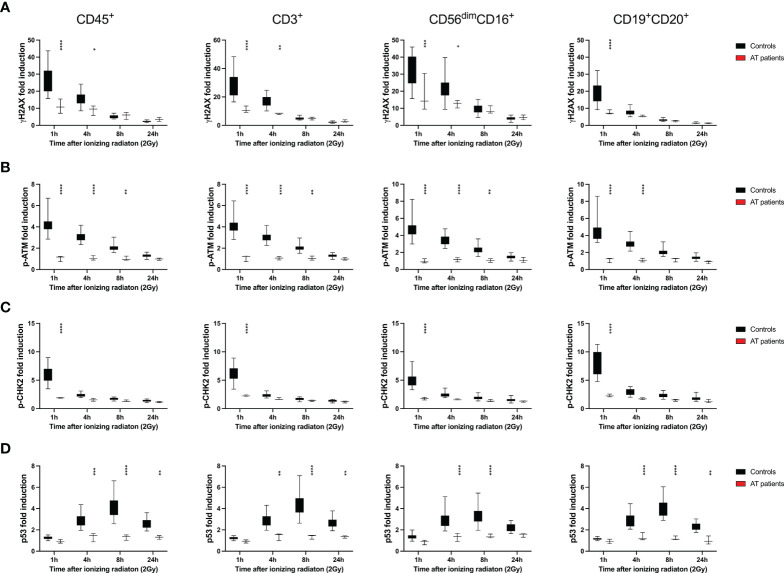
DDR is diminished in all lymphocyte subsets of patients with ataxia telangiectasia. PBMCs obtained from healthy donors and 3 patients with ataxia telangiectasia (AT) were treated with 2Gy ionizing radiation and fixed after 1h, 4h, 8h and 24h. Fold inductions of γH2AX **(A)**, p-ATM **(B)**, p-CHK2 **(C)**, and p53 **(D)** were calculated in CD45^+^, CD45^+^CD3^+^, CD45^+^CD3^-^CD56^dim^CD16^-^, and CD45^+^CD3^-^CD19^+^CD20^+^ lymphocyte subsets, based on mean fluorescence intensities normalized on unirradiated samples. Box plots indicate distribution of fold inductions obtained from 26 healthy donors and 3 AT patients; error bars represent standard deviations. Significance is shown for differences between controls and patients (*p ≤ 0.05, **p ≤ 0.01, ***p ≤ 0.001, ****p ≤ 0.001).

Compared to healthy controls, DDR was significantly impaired in lymphocytes and T, NK and B cell subsets of AT patients (**Figure 9**), which could be used for diagnostic purposes.

## Discussion

The initial process in the response of eukaryotic cells to IR is marked by activation of ATM kinase that is auto-phosphorylated at serine 1981 within minutes after DNA damage (30). Activated ATM kinase phosphorylates a broad range of substrates related to DNA damage repair, cell cycle and apoptosis. Whereas CHK1 is mainly activated be ATR, CHK2 is a major target of ATM and further engaged in cell cycle arrest, DNA repair and apoptosis (31). Another ATM substrate is the tumor suppressor protein p53 balancing survival and apoptotic response. The histone protein H2AX is mainly phosphorylated by ATM, but also other PIKK enzymes such as ATR and DNA-PKcs. Phosphorylation of DDR sensors and transducer proteins can be used as biomarkers for DNA repair capacity to identify individuals with DNA repair defects. However, since these patients often present with lymphopenia and altered lymphocyte subset distributions, differential DNA damage responses of various lymphocyte subsets need to be considered.

In this study, we investigated the DDR of γH2AX (Ser139), p-ATM (Ser1981), p-CHK2 (Thr68) and p53 in naïve and memory T, B and NK lymphocyte subsets of 26 healthy donors by mass cytometry. Our results revealed that γH2AX induction was significantly increased in NK cell subsets compared to T cells, and a diminished response was found in B lymphocytes. Whereas H2AX activation correlated inversely with p-CHK2 and p53 induction in NK cells, we found similar p53 responses in T and B cell subsets 4-8h after DNA damage. P-CHK2 was predominantly activated in B lymphocytes 1h after gamma radiation, and elevated p-ATM response was observed in NK cell subsets. Furthermore, reduced γH2AX induction was associated with increased p-ATM and p53 responses in naïve T and B cells compared to memory subsets.

In order to study DDR of lymphocyte subsets affected by additional types of DNA damage besides IR-induced DNA DSB, PBMCs of 8 healthy donors were exposed to UVC. Compared to IR-treated lymphocytes, UVC exposure resulted in different kinetics of DDR that peaked at later time points of 4h to 8h. Besides differential kinetics, also cellular responses to UVC exposure might be altered. This was studied by comparative analysis of all lymphocyte subsets and revealed differential DDR capacities of T, NK and B cell subsets as observed in response to IR.

Cell counts of viable B lymphocytes significantly decreased in response to IR as shown by proportional alterations of lymphocyte populations. Proportions of naïve CD4^+^ T and naïve B cells also declined 24h after IR, but NK cell subsets did not. These results demonstrate an inverse correlation of DDR capacities with survival potential. However, distinct DDR pathways may be regulated differently and may respond to each as a functional backup, which needs to be addressed in further studies.

Differential survival due to apoptosis induction in response to IR has been studied intensively in lymphocyte subsets (24, 25). Best survival rates and resistance to DNA damage have been reported in NK lymphocytes, and B cells are severely radiosensitive. These findings have been endorsed by differential transcriptional responses to ionizing radiation in lymphocyte subsets (32). P53-dependent proapoptotic genes *BAX* and *TNFRSF10B* are predominantly expressed in CD19^+^ B and CD8^+^ T lymphocytes and to a lesser extend in CD56^+^ NK cells. ATM activation induces transcriptions of genes associated with DNA repair but also lymphocyte development (33, 34). Of note, transcriptional regulations of DNA repair genes differ among lymphocyte subsets. For example, deficient nucleotide excision repair (NER) has been reported in peripheral blood B cells (35).

An additional aspect impacting on differential DDR of cell subsets is chromatin structure. Various modifications of histones at the DNA damage site are involved in the recruitment of DDR-related factors that modulate signaling (36, 37). A highly compacted chromatin has been described in resting T cells compared to proliferating T cells, and changes of histone modification have been observed in the latter (38).

Resting T cells are more radiosensitive than CD3/CD28 stimulated T cells undergoing apoptosis in response to IR, although they do not differ in their DNA repair capacity as shown by comet assays (39). Similar observations have been made in PHA stimulated T cells (40). Our cell cycle studies confirmed that only memory subsets transition to G1 cell cycle phase in response to IL-2, thus resting T cells correspond to naïve T cell subsets. In congruence to our results of increased p-ATM activation in naïve T cells, Heylmann et al. reported reduced ATM expression in stimulated compared to unstimulated PBMCs (39). Reduced γH2AX and 53BP1 foci formation of resting T cells in response to DNA damage has also been described by other groups, although increased numbers of DNA DSBs were found in comet assays (26). These observations suggest deficient repair due to diminished DDR in naïve T cells.

Although DDR is closely related to cell cycle, proliferation does not impact on cell type specific DDR capacities in T and B lymphocytes. Stimulation with IL-2 resulted in activation of memory T cells from G0 to G1 cell cycle subsets and proliferation of NK cells. Subsequently, increased γH2AX and p53 responses were found in NK lymphocytes due to stimulation with IL-2 and proliferation. Although transition to G1 and S cell cycle phases induced DDR intensities, it did not impact on differential DDR capacities as shown by comparative analysis in ki67^-^ and ki67^+^ subsets.

Activation and maturation of T cells is accompanied by a comprehensive change of gene expression and chromatin structure. Transcriptional profiling of exhausted and memory CD8^+^ T cells revealed different expression of factors involved in regulation of immune responses, but also DNA repair genes (41).

In contrast to previous reports indicating restricted p53 activation or phosphorylation to non-quiescent cells (26, 42), we found increased p53 induction in naïve CD4^+^ and CD8^+^ T compared to memory subsets. Despite the strong apoptotic response reported in B lymphocytes, we did not observe an increase in p53 induction in these subsets. However, there is evidence that other apoptotic pathways can be involved besides p53 (26). P53 was predominantly expressed in ki67^+^ CD56^bright^CD16^-^ compared to CD16^+^ NK cell subsets, which was enhanced by UVC exposure.

Interestingly, p-CHK2 was predominantly activated in B cells in response to DNA damage induced both by IR and UVC. Although, CHK2 is required for efficient somatic hypermutation and class switch recombination (43), a particular role in DDR has not been investigated before.

Interindividual differences were particularly identified in NK lymphocytes, as well as transitional B and plasmablast subsets, which impacted on DDR. Maturation of CD56^bright^CD16^-^ to CD56^dim^CD16^+^ NK cells is age dependent and related to viral infections (44), which accounts for interindividual differences in our cohort.

DDR was abrogated in AT patients, although a diminished γH2AX response could be observed. Phosphorylation of H2AX can be partially compensated by other PIKK such as ATR and DNA-PKcs. Phosphorylation of CHK2 selected best between controls and patients, since this biomarker was most effectively activated 1h after IR in PBMCs of healthy controls. CHK2 can also be phosphorylated by DNA-PKcs, another member of the PI3KK family (45), however, this was not observed in our study. Of note, also p-ATM and p53 could not be induced in AT patients.

AT can be diagnosed by newborn screening of T cell receptor excision circles (TRECs) and is a differential diagnosis to severe combined immunodeficiency syndromes (46). Investigation of DDR to IR can be used as a diagnostic tool to identify AT. Induction of γH2AX has been investigated in patients with combined immunodeficiency caused by defects in the NHEJ repair pathway (17–20). Because of differential DDR capacities, diagnostic investigations of DDR on peripheral blood cells should be stratified to different subpopulations.

Although we did not study apoptotic response, impaired survival to IR has been reported for B lymphocytes and resting T cells. Interestingly, particularly B lymphocytes and naïve T cells are diminished in patients with DNA repair deficiencies (16, 46, 47).

A limitation of this study is that all investigations were exclusively performed on cryopreserved material. Although controlled and validated assays are more difficult to perform on fresh PBMCs, cryopreservation may impact on cellular functions such as DDR, which has not been formally tested in this study. Most studies report decreased viability in thawed PBMCs, but no limitations regarding immune profiling (48) and proliferation responses (49). In addition, DNA damage can reliably be analyzed in cryopreserved PBMCs after a 16h period of recovery (50).

Therefore, thawed PBMCs were cultured for 2-4 days to recover from DNA damage.

In summary, our study demonstrates differential DDR in lymphocyte subsets that is not dependent on proliferation. According to previous reports, these observations maybe associated with transcriptional regulation of proapoptotic genes leading to differential survival responses.

## Conclusion

The DDR capacity is differential in lymphocyte subsets independent of cell cycle and proliferation. The strongest DDR can be observed in NK cells, compared to lowest response rates in B lymphocytes, which correlates inversely with DNA damage-related survival. Additionally, naïve T and B cells are characterized by reduced DDR compared to mature memory subsets. DDR is abrogated in all subsets of ATM-deficient lymphocytes obtained from patients affected by AT. Mass cytometry enables comparable investigation of DDR in defined lymphocyte subsets, to which analyses of DNA repair capacities should be stratified.

## Data Availability Statement

The data can be accessed at Harvard Dataverse, https://dataverse.harvard.edu/ doi: 10.7910/DVN/L2DRDV.

## Ethics Statement

The studies involving human participants were reviewed and approved by the ethical review boards of Ulm University (407/16), Technical University of Dresden (TUD) (BO-EK-213052020), and Hannover Medical School (9492-BO-K-2020). Written informed consent to participate in this study was provided by the participants’ legal guardian/next of kin.

## Author Contributions

KF and KS conceptualized, planned and supervised the study. KF performed the experiments and analyzed the data. UB, CK, DV, and CS included AT patients and provided the patient material. SU performed operated analyses using the mass cytometer, which was supported by MH. E-MJ helped with technical advice and gating strategy. AS and K-MD helped with ethical approval of this study. KF and KS wrote the manuscript, which was approved by all co-authors. All authors contributed to the article and approved the submitted version.

## Funding

This study was funded by the German Else-Kroener-Fresenius (EKFS) foundation (2017_A57).

## Conflict of Interest

The authors declare that the research was conducted in the absence of any commercial or financial relationships that could be construed as a potential conflict of interest.

## Publisher’s Note

All claims expressed in this article are solely those of the authors and do not necessarily represent those of their affiliated organizations, or those of the publisher, the editors and the reviewers. Any product that may be evaluated in this article, or claim that may be made by its manufacturer, is not guaranteed or endorsed by the publisher.

## Supplementary Material

Supplementary Figure 1 Gating strategy of lymphocyte subsets analyzed by mass cytometry. Peripheral blood mononuclear cells were fixed and stained as described in material and methods. Live cells were identified by DNA labeling with iridium and cisplatin exclusion staining. T, NK and B cell subsets were analyzed on CD45+ lymphocytes. T cell subsets were characterized as CD3+, CD3+CD4+, CD3+CD8+, CD45RA+CCR7+ (naïve CD4/CD8), CD45RO+CCR7+/- (central and effector memory CD4/CD8). NK lymphocyte subsets defined as CD56brightCD16-, CD56brightCD16+, CD56dimCD16+ were analyzed on CD3- lymphocytes. CD57 was investigated on CD56brightCD16+ and CD56dimCD16+ NK cells. CD3- B lymphocytes were characterized as CD19+CD20+, CD27-IgD+(naïve B), CD27+ (memory B), CD27+IgM+ (unswitched memory B), CD27+IgM-IgGκ+ (class switched memory B IgGκ), CD27+IgM-IgGλ+ (class switched memory B IgGλ), CD27+IgM+IgD+ (Marginal Zone (MZ)-like B), IgM++CD38++ (transitional B), CD27+IgM+IgD- (IgM only B), CD27-IgM-IgD- (atypical memory B), CD19+CD20-CD27+CD38+IgM+ (unswitched plasmablasts), CD19+CD20-CD27+CD38+IgM- (class switched plasmablasts), and CD21lowCD38low B cells.

Supplementary Figure 2 IL-2 stimulation impacts on NK cells counts. PBMCs of 6 healthy donors were cultured in RPMI media without and in presence of 100U/ml human IL-2 for 48h. Cells were irradiated with 2Gy and fixed at indicated time points. Cell counts of viable (Cisplatin-) T, NK, and B lymphocytes (A), naïve and memory CD4+ and CD8+ T-cell subsets (B), CD56brightCD16-, CD56brightCD16+, CD56dimCD16+ NK-cell subsets (C), and naïve and memory B-cell populations (D) cultured with and without IL-2 are compared side at each time point after radiation. Statistical significance was calculated for each lymphocyte population using student’s T test (*p ≤ 0.05).

Supplementary Figure 3 DDR in T-lymphocyte subsets is independent from IL-2 stimulation. PBMCs of 6 healthy donors were cultured in RPMI media without and in presence of 100U/ml human IL-2 for 48h. Cells were irradiated with 2Gy and fixed at indicated time points. Surface markers of lymphocyte subsets and intranuclear DDR biomarkers were assessed by mass cytometry. Mean fluorescence intensities (MFI) of DDR markers γH2AX, p-ATM, p-CHK2 and p53 were calculated in T, NK, and B lymphocyte subsets of unirradiated lymphocytes and 1h, 4h, 8h, 24h following IR with 2Gy. MFIs of DDR markers are compared side-by-side in T-cell subsets cultured with and without IL-2 at each time point following IR. Statistical significance was calculated using Šídák’s multiple comparison test (ns, not significant, *p ≤ 0.05, **p ≤ 0.01, ***p ≤ 0.001, ****p ≤ 0.0001).

Supplementary Figure 4 IL-2 stimulation impacts on DDR in NK-lymphocyte subsets. PBMCs of 6 healthy donors were cultured in RPMI media without and in presence of 100U/ml human IL-2 for 48h. Cells were irradiated with 2Gy and fixed at indicated time points. Surface markers of lymphocyte subsets and intranuclear DDR biomarkers were assessed by mass cytometry. Mean fluorescence intensities (MFI) of DDR markers γH2AX, p-ATM, p-CHK2 and p53 were calculated in T, NK, and B lymphocyte subsets of unirradiated lymphocytes and 1h, 4h, 8h, 24h following IR with 2Gy. MFIs of DDR markers are compared side-by-side in NK-cell subsets cultured with and without IL-2 at each time point following IR. Statistical significance was calculated using Šídák’s multiple comparison test (ns, not significant, *p ≤ 0.05, **p ≤ 0.01, ***p ≤ 0.001, ****p ≤ 0.0001).

Supplementary Figure 5 DDR in B-lymphocyte subsets is independent from IL-2 stimulation. PBMCs of 6 healthy donors were cultured in RPMI media without and in presence of 100U/ml human IL-2 for 48h. Cells were irradiated with 2Gy and fixed at indicated time points. Surface markers of lymphocyte subsets and intranuclear DDR biomarkers were assessed by mass cytometry. Mean fluorescence intensities (MFI) of DDR markers γH2AX, p-ATM, p-CHK2 and p53 were calculated in T, NK, and B lymphocyte subsets of unirradiated lymphocytes and 1h, 4h, 8h, 24h following IR with 2Gy. MFIs of DDR markers are compared side-by-side in B-cell subsets cultured with and without IL-2 at each time point following IR. Statistical significance was calculated using Šídák’s multiple comparison test (ns, not significant, *p ≤ 0.05, **p ≤ 0.01, ***p ≤ 0.001, ****p ≤ 0.0001).

Supplementary Figure 6 IL-2 stimulation does not impact on differential lymphocyte survival rates in response to ionizing radiation. PBMCs of 6 healthy donors were cultured in RPMI media without and in presence of 100U/ml human IL-2 for 48h. Cells were irradiated with 2Gy and fixed at indicated time points. Cell counts of viable (Cisplatin-) T, NK, and B lymphocytes (A), naïve and memory CD4+ and CD8+ T-cell subsets (B), CD56brightCD16-, CD56brightCD16+, CD56dimCD16+ NK-cell subsets (C), and naïve and memory B-cell populations (D) were compared at each time point following radiation. Statistical significance was calculated for each lymphocyte population using Turkey’s multiple comparison test and is shown for unirradiated lymphocytes vs. lymphocytes 24h after IR (ns, not significant, *p ≤ 0.05, **p ≤ 0.01, ***p ≤ 0.001, ****p ≤ 0.0001).

Supplementary Figure 7 Differential IR-induced DDR of lymphocyte subsets is independent from proliferation. PBMCs obtained from 8 healthy donors were irradiated with 2Gy and fixed at indicated time points. Surface markers of lymphocyte subsets and intranuclear DDR biomarkers were analyzed by mass cytometry. Fold inductions of γH2AX, p-ATM, p-CHK2, and p53 were calculated in CD45+ lymphocyte subsets, based on mean fluorescence intensities normalized on unirradiated samples. Analysis of subsets was further distributed by expression of the proliferation marker ki67. Bars show mean values of fold induction; error bars represent standard deviations. Significance calculated by Turkey’s multiple comparison test is shown for naïve T and B cell subsets compared to memory subsets, and immature CD56brightCD16- to mature CD56dimCD16+ NK lymphocytes (*p ≤ 0.05, **p ≤ 0.01, ***p ≤ 0.001, ****p ≤ 0.0001).

Supplementary Figure 8 Cellular proliferation of NK-cell subsets impacts on IR-induced DDR. PBMCs obtained from 8 healthy donors were irradiated with 2Gy and fixed at indicated time points. Surface markers of lymphocyte subsets and intranuclear DDR biomarkers were analyzed by mass cytometry. Fold inductions of γH2AX, p-ATM, p-CHK2, and p53 were calculated in CD45+ lymphocyte subsets, based on mean fluorescence intensities normalized on unirradiated samples. Analysis of subsets was further distributed by expression of the proliferation marker ki67. MFIs of DDR markers in k67+ and ki67- T-, NK-, and B-cell subsets are compared side-by-side at each time point following IR. Statistical significance was calculated using Šídák’s multiple comparison test (ns, not significant, *p ≤ 0.05, **p ≤ 0.01, ***p ≤ 0.001, ****p ≤ 0.0001).

Supplementary Figure 9 Lymphocyte subsets show differential survival response rates to UVC exposure. PBMCs obtained from 8 healthy donors were exposed to 100mJ/m2 UVC and fixed at indicated time points. Cell counts of viable (Cisplatin-) T, NK, and B lymphocytes (A), naïve and memory CD4+ and CD8+ T cell subsets (B), CD56brightCD16-, CD56brightCD16+, CD56dimCD16+ NK cell subsets (C), and naïve and memory B cell populations (D) were compared at each time point following UVC exposure. Statistical significance was calculated for each lymphocyte population using Turkey’s multiple comparison test and is shown for unirradiated lymphocytes vs. lymphocytes 24h after radiation (ns, not significant, *p ≤ 0.05, **p ≤ 0.01, ***p ≤ 0.001, ****p ≤ 0.0001).

Supplementary Figure 10 Differential UVC-induced DDR of lymphocyte subsets is independent from proliferation. PBMCs obtained from 8 healthy donors were treated with 100mJ/m2 UVC and fixed at indicated time points. Surface markers of lymphocyte subsets and intranuclear DDR biomarkers were analyzed by mass cytometry. Fold inductions of γH2AX, p-ATM, p-CHK2, and p53 were calculated in CD45+ lymphocyte subsets, based on mean fluorescence intensities normalized on unirradiated samples. Analysis of subsets was further distributed by expression of the proliferation marker ki67. Bars show mean values of fold induction; error bars represent standard deviations. Significance calculated by Turkey’s multiple comparison test is shown for naïve T and B cell subsets compared to memory subsets, and immature CD56brightCD16- to mature CD56dimCD16+ NK lymphocytes (*p ≤ 0.05, **p ≤ 0.01, ***p ≤ 0.001, ****p ≤ 0.0001).

Supplementary Figure 11 Exposure to UVC results in increased DDR of ki67+ compared to ki67- NK-cell subsets, but DDR is not different in ki67+ and ki67- T- and B-cell populations. PBMCs obtained from 8 healthy donors were exposed to 100mJ/m2 UVC and fixed at indicated time points. Surface markers of lymphocyte subsets and intranuclear DDR biomarkers were analyzed by mass cytometry. Fold inductions of γH2AX, p-ATM, p-CHK2, and p53 were calculated in CD45+ lymphocyte subsets, based on mean fluorescence intensities normalized on unirradiated samples. Analysis of subsets was further distributed by expression of the proliferation marker ki67. MFIs of DDR markers in k67+ and ki67- T-, NK-, and B-cell subsets are compared side-by-side at each time point following IR. Statistical significance was calculated using Šídák’s multiple.

Supplementary Figure 12 DNA damage induced by IR and UVC has similar impact on cell cycle profiles of human lymphocyte subsets. PBMCs obtained from 8 healthy donors were cultured in RPMI media supplemented with 100U/ml human IL-2 for 96h before 2Gy ionizing radiation, or exposure to 100mJ/m2 UVC, respectively. 30min before fixation at indicated time points, IdU was added to cell culture to be incorporated in the DNA of replicating cells. Cell cycle was assessed by mass cytometry using ki67 and IdU to distinguish G0/ki67-IdU-, G1/ki67+IdU- and S/ki67+IdU+ cell cycle phases. Following UVC exposure, also ki67-IdU+ populations could be detected. Bars represent mean percentages of cell cycle phases G0 (grey), G1 (red), S (light blue) and ki67-IdU+ (dark blue) in T, NK and B lymphocyte subsets of unirradiated lymphocytes and 1h, 4h, 8h, 24h after IR or UVC exposure. CD45+ lymphocytes are shown in each panel as internal control. Error bars represent standard deviations.

Supplementary Figure 13 Lymphocyte subsets obtained from patients with AT show no significantly different survival response to ionizing radiation. PBMCs obtained from 3 patients diagnoses with AT were irradiated with 2Gy and fixed at indicated time points. Cell counts of viable (Cisplatin-) T, NK, and B lymphocytes (A), naïve and memory CD4+ and CD8+ T cell subsets (B), CD56brightCD16-, CD56brightCD16+, CD56dimCD16+ NK cell subsets (C), and naïve and memory B cell populations (D) were compared at each time point following radiation. Statistical significance was calculated for each lymphocyte population using Turkey’s multiple comparison test (ns, not significant).

Supplementary Figure 14 DDR is abrogated in patients with ataxia telangiectasia. PBMCs obtained from healthy donors and 3 patients with ataxia telangiectasia (AT) were treated with 2Gy ionizing radiation and fixed after 1h, 4h, 8h and 24h. Expression level of DDR markers γH2AX, p-ATM, p-CHK2 and p53 in all populations at time points analyzed are shown by tSNE Plots. Scale bars on the right-hand side of each panel indicate intensities of DDR markers. The bottom panel represent populations color coded by the legend underneath. One healthy donor (HD) out of 26 is shown together with 3 patients (AT1-3).

Supplementary Figure 15 IR-induced DDR is abrogated in lymphocyte subsets of patients with ataxia telangiectasia. PBMCs obtained from healthy donors and three patients affected by ataxia telangiectasia (AT) were treated with 2Gy ionizing radiation and fixed after 1h, 4h, 8h and 24h. Fold inductions of γH2AX, p-ATM, p-CHK2, and p53 were calculated in CD45+ lymphocyte subsets, based on mean fluorescence intensities normalized on unirradiated samples. Bars show mean values of fold induction; error bars represent standard deviations. There were no significant differences among lymphocyte subsets as calculated by Turkey’s multiple comparison test.
