# Supplementary figures and images for: Differential DNA Damage Response of Peripheral Blood Lymphocyte Populations
Source: Front Immunol. 2021 Sep 14;12:739675. doi: 10.3389/fimmu.2021.739675 (PMC8478158; doi:10.3389/fimmu.2021.739675)

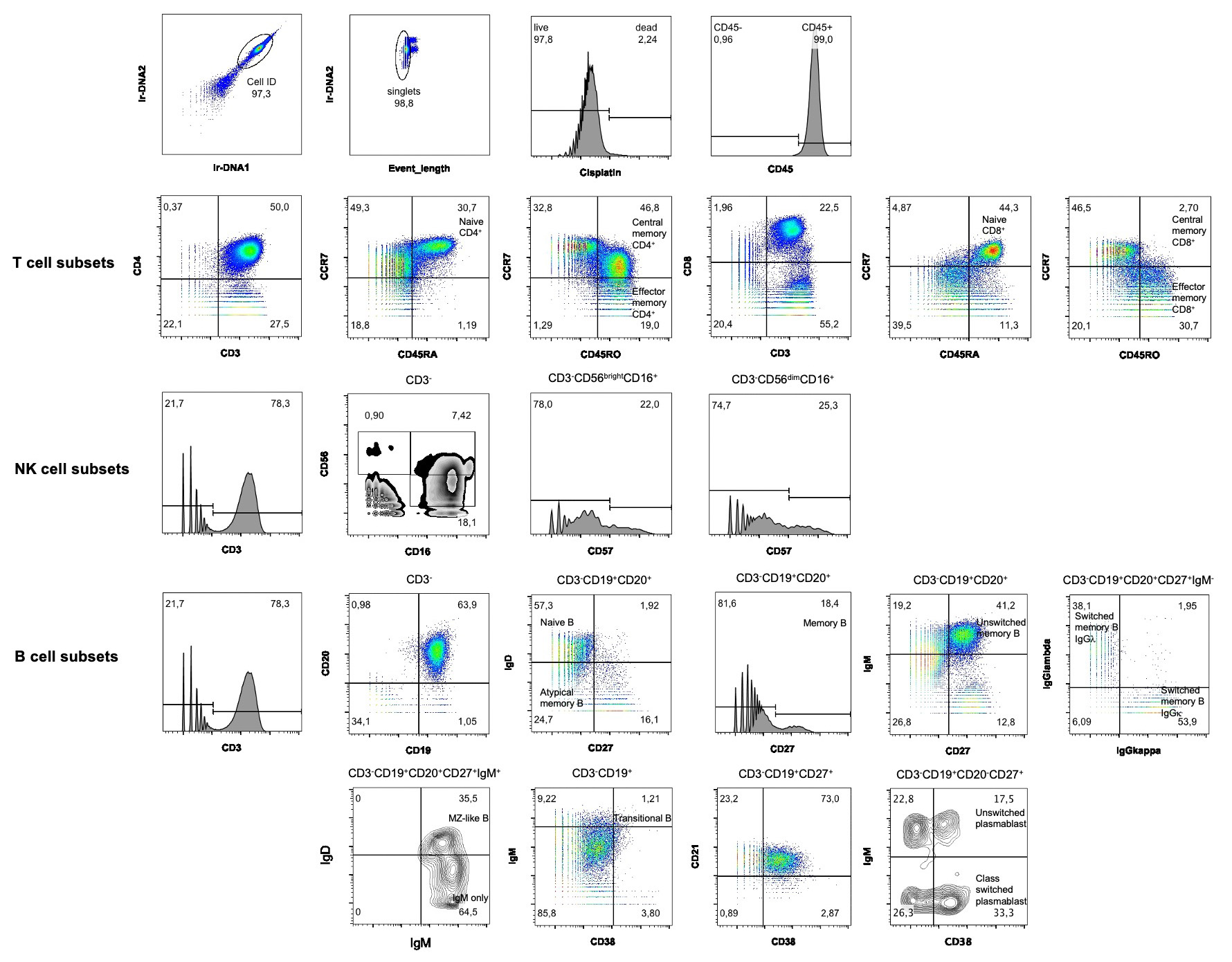

Supplement: Supplementary Figure 1 — Gating strategy of lymphocyte subsets analyzed by mass cytometry. Peripheral blood mononuclear cells were fixed and stained as described in material and methods. Live cells were identified by DNA labeling with iridium and cisplatin exclusion staining. T, NK and B cell subsets were analyzed on CD45+ lymphocytes. T cell subsets were characterized as CD3+, CD3+CD4+, CD3+CD8+, CD45RA+CCR7+ (naïve CD4/CD8), CD45RO+CCR7+/- (central and effector memory CD4/CD8). NK lymphocyte subsets defined as CD56brightCD16-, CD56brightCD16+, CD56dimCD16+ were analyzed on CD3- lymphocytes. CD57 was investigated on CD56brightCD16+ and CD56dimCD16+ NK cells. CD3- B lymphocytes were characterized as CD19+CD20+, CD27-IgD+(naïve B), CD27+ (memory B), CD27+IgM+ (unswitched memory B), CD27+IgM-IgGκ+ (class switched memory B IgGκ), CD27+IgM-IgGλ+ (class switched memory B IgGλ), CD27+IgM+IgD+ (Marginal Zone (MZ)-like B), IgM++CD38++ (transitional B), CD27+IgM+IgD- (IgM only B), CD27-IgM-IgD- (atypical memory B), CD19+CD20-CD27+CD38+IgM+ (unswitched plasmablasts), CD19+CD20-CD27+CD38+IgM- (class switched plasmablasts), and CD21lowCD38low B cells. [file Image_1.jpeg]

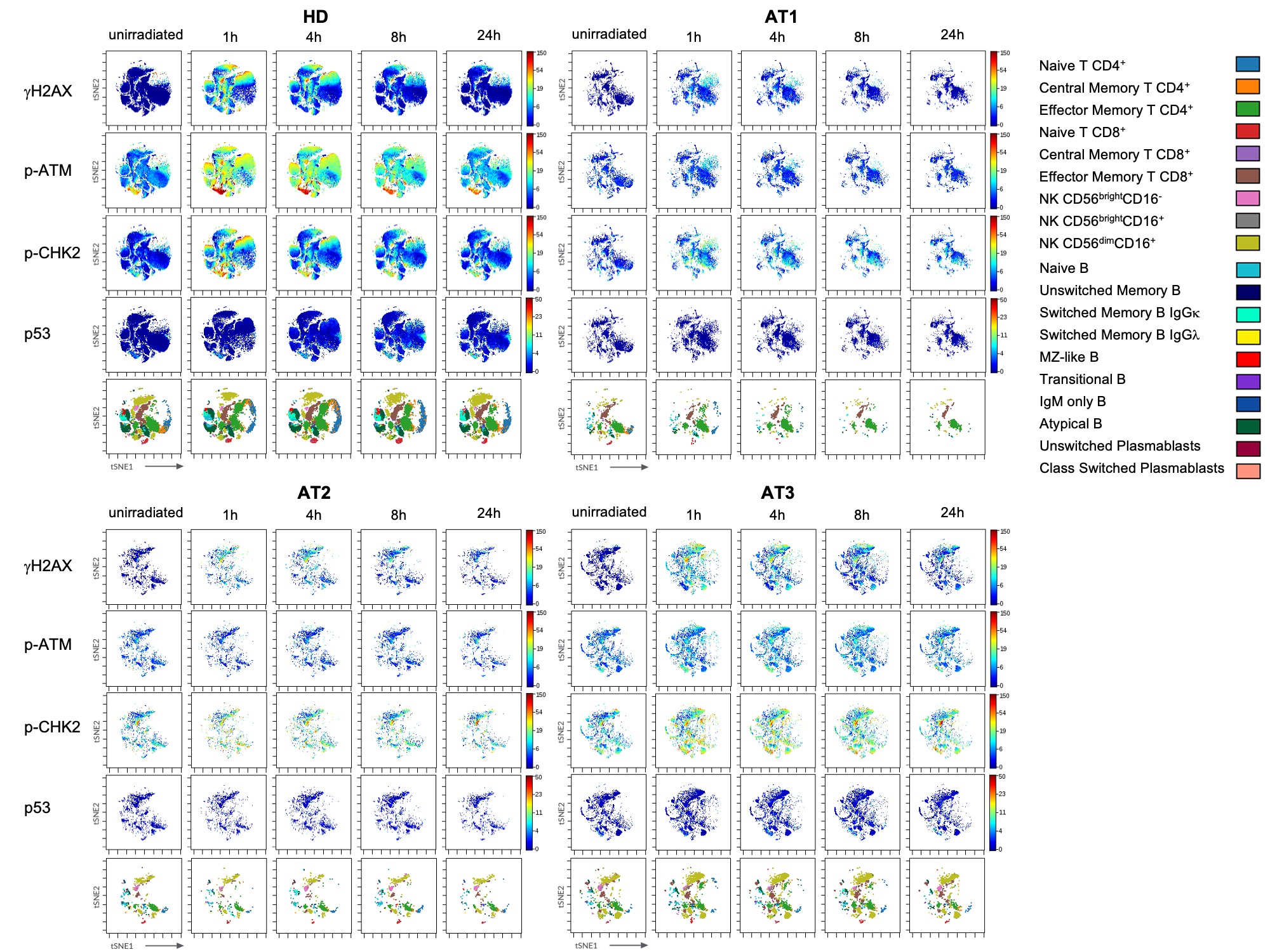

Supplement: Supplementary Figure 14 — DDR is abrogated in patients with ataxia telangiectasia. PBMCs obtained from healthy donors and 3 patients with ataxia telangiectasia (AT) were treated with 2Gy ionizing radiation and fixed after 1h, 4h, 8h and 24h. Expression level of DDR markers γH2AX, p-ATM, p-CHK2 and p53 in all populations at time points analyzed are shown by tSNE Plots. Scale bars on the right-hand side of each panel indicate intensities of DDR markers. The bottom panel represent populations color coded by the legend underneath. One healthy donor (HD) out of 26 is shown together with 3 patients (AT1-3). [file Image_14.jpeg]
